# Modelling the risk of infection transmission due to environmental contamination in hospital single and multi-bed rooms

**DOI:** 10.1186/2047-2994-4-S1-P31

**Published:** 2015-06-16

**Authors:** M-F King, C Noakes, A Sleigh

**Affiliations:** 1 School of Civil Engineering, University of Leeds, Leeds, UK

## Introduction

Aerial dispersion of bioaerosols and subsequent contamination of surfaces is recognised as a potential transmission route for some HCAI infections. Pathogens have been shown to accrue on health-care workers’ (HCW) hands as they touch surfaces [1] and hence can subsequently be transmitted to other patients. This research considers the question: Are single-bed patient rooms more effective than their multi-bed counterparts at reducing this risk?

## Objectives

To link design of current hospital rooms with infection risk from surface contamination and HCW contacts.

## Methods

Computational fluid dynamics simulations, validated through bioaerosol experiments were used to accurately predict spatial distributions of bioaerosol deposition in a single and multi-bed hospital rooms. A Markov chain Monte-Carlo model was developed using the deposition patterns in conjunction with clinical observation of HCW surface contact sequences, to predict the contamination levels of bacteria on HCWs’ hands as they perform routine patient care in the two rooms types.

## Results

Hand colonisation depends on care type, room layout, the number of surface contacts and in particular on the spatial distribution of pathogens between surfaces, which is influenced by ventilation strategy. Contamination on the HCWs' hands decreases monotonically after patient care in a single room due to hand hygiene. During care within multi-bed rooms colonisation levels increase throughout due to the spatial spread of microorganisms contaminating multiple patient surfaces caused by the ventilation strategy. Positioning infectious patients within an unobstructed path between the inlet and outlet diffuser significantly reduces cross contamination to other patients surfaces.

## Conclusion

Results indicate that colonisation levels of HCWs’ hands are likely to be significantly lower after care in single patient rooms than after care in a multi-bed ward and that patient and ventilation design is vitally important in helping curtail the risk of bioaerosol spread.

## Disclosure of interest

None declared.
